# Supplementary material for: A UMLS-based spell checker for natural language processing in vaccine safety
Source: BMC Med Inform Decis Mak. 2007 Feb 12;7:3. doi: 10.1186/1472-6947-7-3 (PMC1805499; doi:10.1186/1472-6947-7-3)
Supplement: Additional file 13 — RAP application directory. Sets up directory for RAP (RDF application for PHP) source code [file 1472-6947-7-3-S13.gz › rap/doc/phpdoc/ontModel/OntProperty.html]

xml version="1.0" encoding="iso-8859-1"?


Docs For Class OntProperty


## Class OntProperty

Description

Description |
Methods (details)

Class encapsulating a property in an ontology.

History: <LI>10-05-2004 : First version of this class.</LI>

- access: public
- author: Daniel Westphal <at d-westphal dot de mail at d-westphal dot de>
- version: V0.9.1

Located in /ontModel/OntProperty.php (line 25)

```
Object
   |
   --Node
      |
      --Resource
         |
         --ResResource
            |
            --OntResource
               |
               --OntProperty
```

Method Summary

Description |
Methods (details)

OntProperty
OntProperty
([string $uri = null])

boolean
addDomain
(object ResResource $resResource)

boolean
addRange
(object ResResource $resResource)

boolean
addSubProperty
(object ResProperty $resProperty)

boolean
addSuperProperty
(object ResProperty $resProperty)

object OntClass
getDomain
()

object OntClass
getRange
()

object OntProperty
getSubProperty
()

object OntProperty
getSuperProperty
()

boolean
hasDomain
(object ResResource $resResource)

boolean
hasRange
(object ResResource $resResource)

boolean
hasSubProperty
(object ResResource $resProperty, [boolean $direct = true])

boolean
hasSuperProperty
(object ResResource $resProperty, [boolean $direct = true])

array
listDomain
()

array
listRange
()

array
listSubProperties
([boolean $direct = true])

array
listSuperProperties
([boolean $direct = true])

boolean
removeDomain
(object ResResource $resResource)

boolean
removeRange
(object ResResource $resResource)

boolean
removeSubProperty
(object ResProperty $resProperty)

boolean
removeSuperProperty
(object ResProperty $resProperty)

void
setDomain
(object ResResource $resResource)

void
setRange
(object ResResource $resResource)

void
setSubProperty
(object ResProperty $resProperty)

void
setSuperProperty
(object ResProperty $resProperty)

Methods

Description |
Methods (details)

Constructor OntProperty (line 34)

Constructor.

You can supply a URI.

- access: public

OntProperty

OntProperty
([string $uri = null])

- string
  $uri

addDomain (line 46)

Add a resource representing the domain of this property.

- access: public

boolean

addDomain
(object ResResource $resResource)

- object ResResource
  $resResource

addRange (line 58)

Add a resource representing the range of this property.

- access: public

boolean

addRange
(object ResResource $resResource)

- object ResResource
  $resResource

addSubProperty (line 70)

Add a sub-property of this property.

- access: public

boolean

addSubProperty
(object ResProperty $resProperty)

- object ResProperty
  $resProperty

addSuperProperty (line 82)

Add a super-property of this property.

- access: public

boolean

addSuperProperty
(object ResProperty $resProperty)

- object ResProperty
  $resProperty

getDomain (line 94)

Answer a OntClass that represents the domain class of this property.

If there is more than one such resource, an arbitrary selection is made.

- access: public

object OntClass

getDomain
()

getRange (line 106)

Answer a OntClass that represents the range class of this property.

If there is more than one such resource, an arbitrary selection is made.

- access: public

object OntClass

getRange
()

getSubProperty (line 118)

Answer a property that is the sub-property of this property.

If there is more than one such property, an arbitrary selection is made.

- access: public

object OntProperty

getSubProperty
()

getSuperProperty (line 134)

Answer a property that is the super-property of this property.

If there is more than one such property, an arbitrary selection is made.

- access: public

object OntProperty

getSuperProperty
()

hasDomain (line 146)

Answer true if the given resource a class specifying the domain of this property.

- access: public

boolean

hasDomain
(object ResResource $resResource)

- object ResResource
  $resResource

hasRange (line 158)

Answer true if the given resource a class specifying the range of this property.

- access: public

boolean

hasRange
(object ResResource $resResource)

- object ResResource
  $resResource

hasSubProperty (line 173)

Answer true if the given property is a sub-property of this property.

If $direct is set to true, only consider the direcly adjacent properties in the property hierarchy

- access: public

boolean

hasSubProperty
(object ResResource $resProperty, [boolean $direct = true])

- object ResResource
  $resProperty
- boolean
  $direct

hasSuperProperty (line 192)

Answer true if the given property is a super-property of this property.

If $direct is set to true, only consider the direcly adjacent properties in the property hierarchy

- access: public

boolean

hasSuperProperty
(object ResResource $resProperty, [boolean $direct = true])

- object ResResource
  $resProperty
- boolean
  $direct

listDomain (line 208)

Answer an array of all of the declared domain classes of this property.

Each element of the iterator will be an OntClass.

- return: of OntClasses
- access: public

array

listDomain
()

listRange (line 220)

Answer an array of all of the declared range classes of this property.

Each element of the iterator will be an OntClass.

- return: of OntClasses
- access: public

array

listRange
()

listSubProperties (line 236)

Answer an array of all the properties that are declared to be sub-properties of this property. Each element of the iterator will be an OntProperty.

If $direct is set to true, only consider the direcly adjacent properties in the property hierarchy

- return: of OntProperties
- access: public

array

listSubProperties
([boolean $direct = true])

- boolean
  $direct

listSuperProperties (line 272)

Answer an array of all the properties that are declared to be super-properties of this property. Each element of the iterator will be an OntProperty.

If $direct is set to true, only consider the direcly adjacent properties in the property hierarchy

- return: of OntProperties
- access: public

array

listSuperProperties
([boolean $direct = true])

- boolean
  $direct

removeDomain (line 300)

Remove the given class from the stated domain(s) of this property.

- access: public

boolean

removeDomain
(object ResResource $resResource)

- object ResResource
  $resResource

removeRange (line 312)

Remove the given class from the stated range(es) of this property.

- access: public

boolean

removeRange
(object ResResource $resResource)

- object ResResource
  $resResource

removeSubProperty (line 324)

Remove the given property from the sub-properties of this property.

- access: public

boolean

removeSubProperty
(object ResProperty $resProperty)

- object ResProperty
  $resProperty

removeSuperProperty (line 336)

Remove the given property from the super-properties of this property.

- access: public

boolean

removeSuperProperty
(object ResProperty $resProperty)

- object ResProperty
  $resProperty

setDomain (line 349)

Assert that the given resource represents the class of individuals that form the domain of this property. Any existing domain statements for this property are removed.

- access: public

void

setDomain
(object ResResource $resResource)

- object ResResource
  $resResource

setRange (line 362)

Assert that the given resource represents the class of individuals that form the range of this property. Any existing range statements for this property are removed.

- access: public

void

setRange
(object ResResource $resResource)

- object ResResource
  $resResource

setSubProperty (line 374)

Assert that this property is super-property of the given property.

Any existing statements for superPropertyOf on prop will be removed.

- access: public

void

setSubProperty
(object ResProperty $resProperty)

- object ResProperty
  $resProperty

setSuperProperty (line 391)

Assert that this property is sub-property of the given property.

Any existing statements for subPropertyOf on prop will be removed.

- access: public

void

setSuperProperty
(object ResProperty $resProperty)

- object ResProperty
  $resProperty

#### Inherited Methods


Inherited From OntResource

> OntResource::OntResource()  
> OntResource::addComment()  
> OntResource::addIsDefinedBy()  
> OntResource::addLabelProperty()  
> OntResource::addProperty()  
> OntResource::addRDFType()  
> OntResource::addSeeAlso()  
> OntResource::asClass()  
> OntResource::asIndividual()  
> OntResource::asOntProperty()  
> OntResource::getComment()  
> OntResource::getInstanceRdfType()  
> OntResource::getIsDefinedBy()  
> OntResource::getLabelProperty()  
> OntResource::getPropertyValue()  
> OntResource::getRDFType()  
> OntResource::getSeeAlso()  
> OntResource::getVocabulary()  
> OntResource::hasComment()  
> OntResource::hasLabelProperty()  
> OntResource::hasRDFType()  
> OntResource::hasSeeAlso()  
> OntResource::isDefinedBy()  
> OntResource::listComments()  
> OntResource::listIsDefinedBy()  
> OntResource::listLabelProperties()  
> OntResource::listProperty()  
> OntResource::listRDFTypes()  
> OntResource::listSeeAlso()  
> OntResource::removeComment()  
> OntResource::removeDefinedBy()  
> OntResource::removeLabelProperty()  
> OntResource::removeProperty()  
> OntResource::removeRDFType()  
> OntResource::removeSeeAlso()  
> OntResource::setComment()  
> OntResource::setInstanceRdfType()  
> OntResource::setIsDefinedBy()  
> OntResource::setLabelProperty()  
> OntResource::setPropertyValue()  
> OntResource::setRDFType()  
> OntResource::setSeeAlso()  
> OntResource::setVocabulary()

Inherited From ResResource

> ResResource::ResResource()  
> ResResource::addProperty()  
> ResResource::equals()  
> ResResource::getAssociatedModel()  
> ResResource::getIsAnon()  
> ResResource::getProperty()  
> ResResource::hasProperty()  
> ResResource::listProperties()  
> ResResource::removeAll()  
> ResResource::removeProperties()  
> ResResource::setAssociatedModel()  
> ResResource::setIsAnon()  
> ResResource::setURI()

Inherited From Resource

> Resource::Resource()  
> Resource::equals()  
> Resource::getLabel()  
> Resource::getLocalName()  
> Resource::getNamespace()  
> Resource::getURI()  
> Resource::toString()

Inherited From Object

> Object::toString()

Documentation generated on Thu, 7 Jul 2005 13:42:25 +0200 by phpDocumentor 1.3.0RC3
